# Comparing Interferon-Gamma Release Assays to Tuberculin Skin Test in Thai Children with Tuberculosis Exposure

**DOI:** 10.1371/journal.pone.0105003

**Published:** 2014-08-14

**Authors:** Hong-Van Tieu, Piyarat Suntarattiwong, Thanyawee Puthanakit, Tawee Chotpitayasunondh, Kulkanya Chokephaibulkit, Sunee Sirivichayakul, Supranee Buranapraditkun, Patcharawee Rungrojrat, Nitiya Chomchey, Simon Tsiouris, Scott Hammer, Vijay Nandi, Jintanat Ananworanich, on behalf of the Thai TB Px study group

**Affiliations:** 1 Laboratory of Infectious Disease Prevention, Lindsley F. Kimball Research Institute, New York Blood Center, New York, New York, United States of America; 2 Division of Infectious Diseases, Department of Medicine, Columbia University Medical Center, New York, New York, United States of America; 3 Queen Sirikit National Institute of Child Health, Bangkok, Thailand; 4 Department of Pediatrics, Faculty of Medicine, Chulalongkorn University, Bangkok, Thailand; 5 HIV Netherlands Australia Thailand Research Collaboration (HIV-NAT), The Thai Red Cross AIDS Research Center, Bangkok, Thailand; 6 Department of Pediatrics, Faculty of Medicine, Siriraj Hospital, Mahidol University, Bangkok, Thailand; 7 Department of Medicine, Faculty of Medicine, Chulalonglongkorn University, Bangkok, Thailand; 8 SEARCH, The Thai Red Cross AIDS Research Center, Bangkok, Thailand; 9 U.S. Military HIV Research Program, Walter Reed Army Institute of Research, Silver Spring, Maryland, United States of America; McGill University, Canada

## Abstract

**Background:**

Data on the performance of interferon-gamma release assays (IGRAs), QuantiFERON TB Gold In-tube (QFNGIT) and T-Spot.TB, in diagnosing tuberculosis (TB) are limited in Southeast Asia. This study aims to compare the performances of the two IGRAs and TST in Thai children with recent TB exposure.

**Methods:**

This multicenter, prospective study enrolled children with recent exposure to active TB adults. Children were investigated for active TB. TST was performed and blood collected for T-Spot.TB and QFNGIT.

**Results:**

158 children were enrolled (87% TB-exposed and 13% active TB, mean age 7.2 years). Only 3 children had HIV infection. 66.7% had TST≥10 mm, while 38.6% had TST≥15 mm. 32.5% had positive QFNGIT; 29.9% had positive T-Spot.TB. QFNGIT and T-Spot.TB positivity was higher among children with active TB compared with TB-exposed children. No indeterminate IGRA results were detected. No statistically significant differences between the performances of the IGRAs and TST at the two cut-offs with increasing TB exposure were detected. Concordance for positive IGRAs and TST ranged from 42–46% for TST≥10 mm and 62–67% for TST≥15 mm. On multivariable analyses, exposure to household primary/secondary caregiver with TB was associated with positive QFNGIT. Higher TB contact score and active TB were associated with positive T-Spot.TB.

**Conclusions:**

Both QFNGIT and T-Spot.TB performed well in our Thai pediatric study population. No differences in the performances between tests with increasing TB exposure were found. Due to accessibility and low cost, using TST may more ideal than IGRAs in diagnosing latent and active TB in healthy children in Thailand and other similar settings.

## Background

Tuberculosis (TB) is a major cause of childhood morbidity and mortality globally, [1] with children who have contact with an adult with active pulmonary TB experiencing an elevated risk of infection. [2] For example, children under the age of one year have a 50–60% risk of progression to active TB during the first year of infection, [3] with the risk increased with HIV co-infection. [4] In 2012 Thailand ranked among 22 high TB burden countries, with an estimated 80,000 people developing TB annually. [5] Thirteen percent of TB cases in Thailand are HIV-associated, and approximately 2,300 TB cases occur in children each year. Among children exposed to adults with active TB in a Thai study, 7% developed active TB while 65% had latent TB; 14% of the children were HIV-infected [6].

The diagnosis of TB in children is often challenging. [2], [7] In most countries with endemic TB, tuberculin skin test (TST) is used to diagnose latent TB. However, TST has low sensitivity and specificity, resulting in imperfect estimation of TB infection risk. [8]–[10] Children previously vaccinated with Bacille Calmette-Guérin (BCG) or infected with nontuberculous mycobacteria frequently have false positive TST reactions. In addition, children who are malnourished or have HIV or other immunocompromised disease often have false negative TST reactions. [3], [11] Data on the diagnosis of latent and active TB in children using interferon-gamma release assays (IGRAs), which are based on *M. tuberculosis*-specific antigens, are accruing. [8]–[11] Potential benefits of IGRAs compared to TST include higher specificity and increased compliance (unlike TST, IGRAs do not require a follow-up visit for the test reading). [12] Because IGRAs use more specific Mycobacterium antigens (ESAT-6, CFP-10, and TB7.7) encoded by genes in the region of 1 (RD1) of the M. tuberculosis genome, they are not affected by cross-reactivity with BCG vaccine strains of M. bovis, [11], [13] which provides another advantage of IGRAs over TST in countries with high BCG vaccination coverage.

A 2007 meta-analysis showed that the interferon-gamma release assays, QuantiFERON-TB Gold In-tube (QFNGIT) (a whole blood assay, Cellestis Limited, Carnegie, Victoria, Australia) and T-Spot.TB (a T-cell-based assay, Oxford Immunotec, Oxford, UK), had higher sensitivity than TST (66% and 62%, respectively, vs. 55%) in detecting latent TB in children. [14] A more recent meta-analysis [8] of studies among children in low, moderate, and high income countries observed slightly higher sensitivity and specificity of IGRAs compared with TST in active TB (pooled sensitivity of 80% for TST vs. 83–84% for the IGRAs; pooled specificity of 85% for TST vs. 91–94% for the IGRAs), although the confidence intervals for both sensitivity and specificity overlapped. A trend towards lower sensitivity for all three tests was noted in study samples with high BCG vaccination coverage in the meta-analysis. Another meta-analysis found that in low-income countries, IGRAs were not superior to TST. [10] Only one study, conducted in Papua New Guinea, has been performed assessing the utility of QFNGIT in latent and active TB diagnosis in children in Southeast Asia;[15] however, no studies involving a three-way comparison of the two IGRAs and TST have been previously completed in Southeast Asia [10].

Using baseline data from our prospective, multi-site cohort study in Bangkok, Thailand, we compared the performances of the IGRAs (T-Spot.TB, QuantiFERON-TB Gold In-tube) and TST at two different cut-off thresholds (10 mm and 15 mm) in Thai children who had recent exposure to an adult index case with TB by determining what test correlates better with increasing TB exposure. We also assessed concordance and discordance between these tests. In addition, we evaluated factors such as age, nutritional and immune status, type and duration of contact with index TB case, and TB infection status on the children’s response to the IGRAs and TST at the baseline visit. Both 10 mm and 15 mm thresholds were examined in this study (1) to add relevance to the Thai clinical setting since Thai guidelines recommend treatment for latent TB in children aged 5–16 years with TST≥15 mm [16], and (2) to add relevance of the study findings to international settings in which different TST cut-offs are used.

## Materials and Methods

This prospective cohort study was conducted between September 2009 and December 2011 at three sites in Bangkok, Thailand: Queen Sirikit National Institute of Child Health, HIV Netherlands Australia Thailand (HIV-NAT) Clinic/Chulalongkorn Hospital, and Siriraj Hospital.

### Study Population

Children between the ages of 2 months and 16 years with recent exposure (defined as having lived with and/or having had close contact with) to adults with active pulmonary TB (confirmed by positive AFB stain, PCR for TB, or TB culture), with or without extrapulmonary TB manifestations, during the past year were referred to the three study sites for eligibility screening. Interested subjects were then screened for eligibility, and all parents or caregivers of the eligible children were asked to provide written informed consent after presentation and explanation of the study and consent form. Child assent was obtained from children ≥7 years of age. Children were excluded if they and/or their caregivers refused study participation, if they were receiving anti-TB medications for TB disease (including isoniazid [INH] for latent TB), or if they had recently been diagnosed with active TB.

### Study Procedures

At the baseline visit, the children were evaluated for active TB with the following components at baseline visit: (1) standardized patient history (contact and medical history, symptom-based questionnaire), (2) BCG vaccination history, TB exposure and TST history, (3) standardized physical examination, (4) chest x-ray, (4) abdominal ultrasound for subjects with abdominal symptoms and signs, (5) for those with active symptoms or abnormal chest x-ray: at least 3 sputum or early morning gastric aspirate specimens (if child was unable to provide sputum specimens) for acid fast bacilli (AFB) smear and culture, (6) other tissue specimens for AFB smear and culture as clinically indicated.

In this study, for HIV status, in accordance with Thai HIV treatment guidelines that recommend HIV antibody testing for every pregnant woman during antenatal care, children who were otherwise healthy, had no history of material positive HIV serostatus, and had no HIV-related signs and symptoms (such as hepatosplenomegaly, recurrent bacterial and viral infections, opportunistic infections) were presumed to have negative HIV status. The other enrolled children received HIV antibody or viral load PCR testing.

At the baseline visit, the children had a TST (0.1 ml solution or 10 international units of tuberculin purified protein derivative) implanted on the forearm followed by result reading by trained health care personnel in 48–72 hours, in accordance with Thai national guidelines. The size of TST induration was determined by measuring the maximum width (or transverse diameter) of an indurated lesion.

The children had whole blood and peripheral blood mononuclear cells collection for the interferon-gamma release assays (T-Spot.TB and QFNGIT). The blood samples were sent on the same day of collection to the laboratory of the Department of Allergy and Immunology, Faculty of Medicine, Chulalongkorn University for testing according to the manufacturers’ instructions using positive and negative controls. [17], [18] Results for T-Spot.TB were reported as positive, negative, or indeterminate, [18] while results for QFNGIT were reported as positive, negative, or indeterminate according to the manufacturers’ guidelines. [17] Positive cutoff values for the tests were defined using the manufacturers’ standard guidelines. Study investigators, site coordinators, and clinicians were blinded to the results of the IGRAs until the study had completed enrollment and 9-month follow-up.

### TB Contact Score

A TB contact score was computed using an algorithm modified from a previous study [3] (Table 1). The TB contact score was weighted based on the relationship of the TB index case to the child, type and duration of exposure to index case, and the infectivity of the index case based on sputum AFB positivity, and could range from 6 to 19. A TB contact score for each child was calculated. A mean TB contact score was computed for the overall cohort and for TB-exposed and active TB cases.

**Table 1:** Mycobacterium TB contact score.

Variable	Weight assigned
TB index case relationship to child	
Primary caregiver in household with TB	4
Secondary caregiver in household with TB	3
Relative/other contact in household with TB	2
Non-household TB contact	1
Type of exposure to TB index case	
Sleeps in same room	3
Lives and sleeps in same house	2
Lives and sleeps in different house	1
Duration of average contact per day withTB index case	
0–3 hours	1
4–7 hours	2
8–11 hours	3
≥12 hours	4
Duration of contact with index case in last12 months (months)	
0–3 months	1
3.1–6 months	2
6.1–9 months	3
9+ months	4
Index TB case history	
Sputum AFB negative	2
Sputum AFB positive	4
Total contact score (maximum = 19)	

### TB Case Definitions

The children were classified into the following categories independent of results of TST and IGRAs: active TB (definite, probable, or possible TB) and TB-exposed. Case definitions for these categories were pre-determined by the investigators prior to study enrollment. A definite TB case required culture evidence of *Mycobacterium tuberculosis* in at least one clinical specimen or AFB smear positive in at least 2 clinical specimens. A child was classified as having probable TB if he/she had any of the following: (1) caseating granuloma on tissue biopsy, (2) spinal gibbus, or (3) AFB smear positive in 1 clinical specimen *plus* at least 1 of the following: (a) chest x-ray findings suggestive of active TB, (b) cervical lymphadenopathy with sinus, (c) abdominal ascites or mass with associated lymphadenopathy on radiographic imaging, (d) clinical symptoms of meningitis with CSF findings consistent with TB meningitis or neuroradiographic finding of basal meningeal enhancement or tuberculoma, and (e) erythema nodosum or phylectenular conjunctivitis with chest x-ray evidence of primary TB. Children were classified as having possible TB if the above criteria for definite and probable TB were not fulfilled and active TB could not be excluded. A child was categorized as being TB-exposed if he/she met the following criteria: (1) asymptomatic, (2) had close contact with an active pulmonary TB (with or without extrapulmonary involvement) adult case during the past year, and (3) no active TB disease (definite, probable, possible TB) confirmed.

### TB Treatment Course

Children in the study were treated according to Thai national TB program guidelines. Children who met the following criteria were started on INH chemoprophylaxis for 9 months: children aged <5 years and HIV-infected children with exposure to active pulmonary TB irrespective of TST result; and children aged 5–16 years with TST≥15 mm or recent converter were started on INH chemoprophylaxis for 9 months. For children aged 5–16 years with TST between 10 to 15 mm, INH could be considered based on the judgment of the healthcare provider. [16] If the index adult case was known to have INH drug resistance, then the child received rifampicin for 4 months. If the adult index case was known to have multi-drug resistant TB, the choice of latent TB medication was based on consultation with the study’s principal investigators. Subjects who met the study definitions of definite, probable, or possible TB were treated according to the Thai national TB program guidelines with a standard four-drug regimen consisting of INH, rifampicin, ethambutol, and pyrazinamide for at least 6 months.

### Statistical Analysis

Data were analyzed using SAS version 9.3 (SAS Institute Inc., Cary, NC, USA). The proportion of children with positive IGRA and TST results at the baseline month 0 study visit was compared using Chi-square test and Fisher’s exact test. We calculated the Spearman correlation coefficient between positivity of each test (QFNGIT, TSpot.TB, and TST at 10 mm and 15 mm cutoffs) and graded TB exposure based on the TB contact score (divided into 4 categories based on the mean TB contact score) for the total sample (TB-exposed and active TB) and for TB-exposed only. Next, we computed the odds ratio (OR) and 95% confidence interval (CI) of test positivity for each test for each TB exposure gradient category, with the lowest TB contact score of 8–10 as the reference group. We then plotted regression lines of the natural logs of the ORs for each test for each TB exposure gradient relative to the TB contact score of 8–10. We pre-determined that a test with a steeper regression slope (i.e., larger change in the natural log of OR of test positivity with increasing TB exposure) would have superior performance in detecting infection with TB. [8] Concordance and discordance between TST at the two cut-off values and IGRAs for the overall cohort, TB-exposed cases, and active TB cases was assessed using the kappa statistic. Bivariable logistic regression was performed to determine whether sociodemographic variables, study site, TB index case exposure type and duration, TB contact score, HIV status, nutritional status (WHO weight-for-age Z-score), and TB infection status were associated with the following at the baseline visit: (1) having a positive QFNGIT result, (2) having a positive T-Spot.TB result, (3) having both a positive QFNGIT and a positive T-Spot.TB result, (4) having a TST≥10 mm, and (5) having a TST≥15 mm. Variables with p-values<0.05 were then selected for the multivariable logistic regression models using a forward selection process. All analyses were two-sided with alpha = 0.05.

The institutional review boards at Chulalongkorn University, Queen Sirikit National Institute of Child Health, Siriraj Hospital, Columbia University, and New York Blood Center approved the study.

## Results

A total of 158 children were enrolled in the study, with 137 (87%) meeting the study’s case definition for TB-exposed and 21 (13%) meeting the definition for active TB (2 definite, 6 possible, and 13 probable TB). An overall summary of the baseline characteristics of the cohort and a comparison by TB case classification (TB-exposed vs. active TB) is presented in Table 2. Overall mean age of the children was 7.2 years (SD 4.2), with those with active TB being younger than TB-exposed children. Only 3 (1.9%) were HIV-positive; 11 (7.1%) were HIV-negative; and 142 (91.0%) were not offered HIV testing due to the lack of history of parental HIV infection and clinical symptoms consistent with HIV. Most children, 96.8%, reported BCG vaccination at birth, with 76.4% of the children had a BCG scar present on physical exam. Only 1 child had received systemic steroids in the 4 weeks prior to study enrollment. Compared with TB-exposed cases, children with active TB were more likely to have been exposed to a secondary caregiver in the household with TB (42.9% vs. 17.9%) and less likely have been exposed to a primary caregiver or relative or other contact in the household with TB (28.6% vs. 37.3% and 9.5% vs. 37.3%, respectively, p = 0.004). However, there were no significant differences in type of exposure and duration of contact between the adult TB index case and child by TB infection status. The mean TB contact score was 12.6 (SD 2.0) for the overall cohort, with no difference detected between active TB and TB-exposed cases.

**Table 2:** Baseline Characteristics, TST, and IGRA Results of Thai Pediatric Cohort, N = 158.

	Total(N = 158)	TB-exposed(N = 137)	Active TB(Definite, possible,probable TB)(N = 21)	P-value
Baseline Characteristic, N (%)				
Age, mean (SD) (years)	7.2 (4.2)	7.6 (4.3)	4.7 (1.7)	<0.001
Gender				0.59
Male	81 (51.6)	69 (50.7)	12 (57.1)	
Female	76 (48.4)	67 (49.3)	9 (42.9)	
HIV serostatus				0.75†
Positive	3 (1.9)	3 (2.2)	0 (0.0)	
Negative	11 (7.1)	9 (6.6)	2(10.0)	
Unknown*	142 (91.0)	124 (91.2)	18 (90.0)	
History of prior latent or active TB	5 (3.2)	4 (2.9)	1 (4.8)	0.52†
History of BCG vaccination				NA
Yes	153 (96.8)	132 (96.4)	21 (100.0)	
No	1 (0.6)	1 (0.7)	0 (0.0)	
Unknown	4 (2.5)	4 (2.9)	0 (0.0)	
BCG scar on physical exam	113 (76.4)	95 (73.6)	18 (94.7)	0.05†
Received any steroids during the last 4 weeks				NA
Yes	1 (0.6)	0 (0.0)	1 (4.8)	
No	157 (99.4)	137 (100.0)	20 (95.2)	
Type and Duration of Exposureto TB Index Case				
TB index case relationship to child				0.004†
Primary caregiver in household with TB	56 (36.1)	50 (37.3)	6 (28.6)	
Secondary caregiver in household with TB	33 (21.3)	24 (17.9)	9 (42.9)	
Relative/other contact in household with TB	52 (33.6)	50 (37.3)	2 (9.5)	
Non-household TB contact	14 (9.0)	10 (7.5)	4 (19.1)	
Type of exposure to TB index case				0.73
Sleeps in same room	72 (46.5)	63 (47.0)	9 (42.9)	
Lives and sleeps in same house	62 (40.0)	54 (40.3)	8 (38.1)	
Lives and sleeps in different house	21 (13.6)	17 (12.7)	4 (19.1)	
Duration of average contactper day with TB index case (hours)				0.82
0–3	2 (1.3)	2 (1.5)	0 (0.0)	
4–7	41 (26.8)	34 (25.8)	7 (33.3)	
8–11	26 (17.0)	24 (18.2)	2 (9.5)	
12+	84 (54.9)	72 (54.6)	12 (57.1)	
Duration of contact with indexcase in last 12 months (months)	7.0 (3.9)	6.9 (4.0)	7.7 (3.7)	0.38
Index TB case history				
Sputum AFB negative	7 (4.5)	7 (5.2)	0 (0.0)	0.60†
Sputum AFB positive	145 (92.4)	125 (91.9)	20 (95.2)	1.00†
Mycobacterium TB culture positive	7 (4.5)	5 (3.7	2 (9.5)	0.24†
TB contact score				0.27
8–12	61 (41.2)	55 (43.0)	6 (30.0)	
13+	87 (58.8)	73 (57.0)	14 (70.0)	
Child’s Clinical Symptoms, Diagnostics, and Treatment Course				
Mean weight for age Z-score (SD)Z-score ≤ −2.00 SD	–0.05 (1.29)11 (7.50)	–0.10 (1.28)9 (7.0)	0.27 (1.32)2 (10.5)	0.250.64†
Clinical symptoms and signs				NA
Cough	30 (19.0)	25 (18.3)	5 (23.8)	
Fever	37 (23.4)	29 (21.2)	8 (38.1)	
Unexplained weight loss	6 (4.1)	4 (3.2)	2 (10.5)	
Dyspnea	5 (3.2)	2 (1.5)	3 (14.3)	
Abdominal pain	4 (2.6)	2 (1.5)	2 (9.5)	
Abnormal lung exam	5 (3.2)	1 (0.8)	4 (19.1)	
Enlarged lymph nodes	3 (1.9)	2 (1.5)	1 (4.8)	
Abdominal ascites/distention	1 (0.7)	0 (0.0)	1 (4.8)	
Meningismus	1 (0.7)	0 (0.0)	1 (4.8)	
Chest radiograph findings				NA
Normal	100 (63.7)	98 (72.1)	2 (9.5)	
Hilar infiltrate(s) with lower lobe infiltrate(s)	4 (2.5)	2 (1.5)	2 (9.5)	
Hilar infiltrate(s) with interstitial infiltrates	1 (0.6)	0 (0.0)	1 (4.8)	
Hilar infiltrate(s) with lymph node disease	2 (1.3)	0 (0.0)	2 (9.5)	
Interstitial infiltrate(s) with/without lymph node disease	2 (1.3)	2 (1.5)	0 (0.0)	
Infiltrate(s) with pleural effusion				
Other infiltrate(s)	1 (0.6)	0 (0.0)	1 (4.8)	
Lymph node disease only	39 (24.7)	28 (20.6)	11 (52.4)	
Other	4 (2.5)	4 (2.9)	0 (0.0)	
Missing/not done	4 (2.5)	2 (1.5)	2 (9.5)	
TB microbiologic confirmation (N = 3)				NA
AFB sputum or gastric lavage culture positive	2 (1.3)	0 (0.0)	2 (9.5)	
M. TB PCR sputum or gastric lavage positive	0 (0.0)	0 (0.0)	0 (0.0)	
AFB blood culture positive for M. TB	1 (0.6)	0 (0.0)	1 (4.8)	
Child’s treatment course				NA
No treatment	21 (13.6)	20 (15.0)	1 (4.8)	
Active TB disease treatment	20 (13.0)	0 (0.0)	20 (95.2)	
INH prophylaxis for latent TB	113 (73.4)	113 (85.0)	0 (0.0)	
TST and IGRA Results, Baseline Month 0 Visit				
Tuberculin skin test				
≥10 mm	102 (66.7)	88 (66.7)	14 (66.7)	1.00
≥15 mm	59 (38.6)	48 (36.4)	11 (52.4)	0.16
QFNGIT result				0.04
Positive	51 (32.5)	40 (29.4)	11 (52.4)	
Negative	106 (67.5)	96 (70.6)	10 (47.6)	
Indeterminate	0 (0.0)	0 (0.0)	0 (0.0)	
T-Spot.TB result				0.02
Positive	47 (29.9)	36 (26.5)	11 (52.4)	
Negative	110 (70.1)	100 (73.5)	10 (47.6)	
Equivocal/borderline	0 (0.0)	0 (0.0)	0 (0.0)	

† Fisher’s exact.

NA: not applicable AFB: acid fast bacilli.

IGRA: Interferon gamma release assays QFNGIT: QuantiFERON Gold In-Tube.

*HIV testing was not offered because of lack of history of parental HIV infection and clinical signs/symptoms consistent with HIV infection.

Two-thirds of the overall cohort had a TST≥10 mm induration at the baseline visit, while 38.6% had a TST≥15 mm induration; no statistically significant difference was noted by TB infection status. Overall, 32.5% of the children had a positive QFNGIT result, while 29.9% had a positive T-Spot.TB result. QFNGIT positivity was higher among children with active TB compared with TB-exposed children (52.4% vs. 29.4%, p = 0.04). Similarly, T-Spot.TB positivity was higher among children with active TB than TB-exposed children (52.4% vs. 26.5%, p = 0.02). No indeterminate QFNGIT results and no equivocal/borderline T-Spot.TB results were detected in the cohort.

Table 3 shows the correlation coefficient of the various tests for *M. tuberculosis* with graded TB contact exposure based on the TB contact score for the total sample (TB-exposed and active TB) and for TB-exposed only. For the total sample, the correlation coefficient between TST≥10 mm and TB contact exposure was 0.27; TST≥15 mm 0.24; QFTGIT 0.31; and T-Spot.TB 0.28. For TB-exposed only, the correlation coefficient between TST≥10 mm and TB contact exposure was 0.22; TST≥15 mm 0.16; QFTGIT 0.25; and T-Spot.TB 0.21.

**Table 3:** Correlation of Tests for *M. tuberculosis* with Graded Exposure to Tuberculosis.

	Total						TB-Exposed Only					
	Exposure category based on TB contact score						Exposure category based on TB contact score					
	8–10	11–12	13–14	15–16	Spearman CorrelationCoefficient	P-value	8–10	11–12	13–14	15–16	Spearman CorrelationCoefficient	P-value
TST≥10 mm	7/21	26/40	44/60	20/25	0.27	0.001	6/17	26/38	36/51	16/20	0.22	0.014
TST≥15 mm	2/21	15/40	25/60	14/25	0.24	0.003	2/17	15/38	18/51	10/20	0.16	0.076
QFTGIT	2/21	8/40	25/61	13/25	0.31	<0.001	2/17	8/38	17/52	10/20	0.25	0.005
T-Spot. TB	2/21	8/40	23/61	12/25	0.28	<0.001	2/17	8/38	15/52	9/20	0.21	0.017

**Figure 1:**
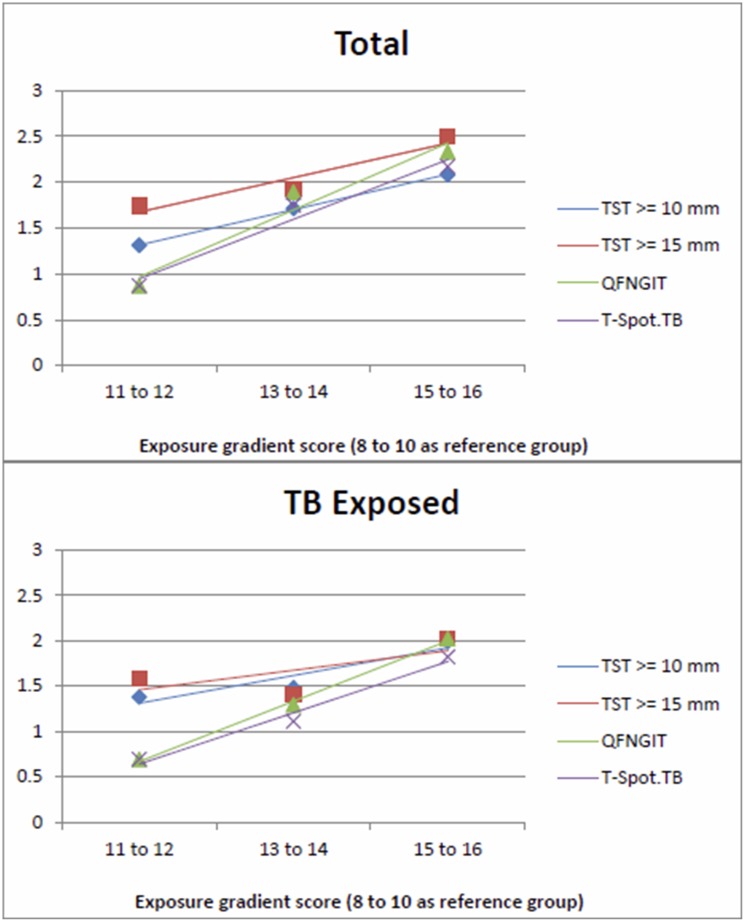
The regression lines of the natural logs of the odds ratios (y-axis) for TST≥10 mm, TST≥15 mm, QFNGIT, and T-Spot. TB for each TB exposure gradient category (x-axis), with TB contact score of 8–10 as the reference group.

In Table 4, the ORs and 95% CIs for positivity of each test for *M. tuberculosis* for each exposure gradient category were compared, with TB contact score of 8–10 as the reference group, for the total sample and for TB-exposed children only. There were no statistical significant differences between the tests due to overlapping CIs for both the total sample and for TB-exposed only. In addition, the 95% CIs for all the tests were found to be wide. Figure 1, which depicts the regression lines of the beta estimates for each test for each TB exposure gradient category relative to the lowest exposure group, shows that for each test, the odds of test positivity (slope) increases as the TB exposure increases. Figure 1 reflects the finding that there were no significant differences between the performances of the tests with increase in TB exposure.

**Table 4:** Association between Tests for *M. tuberculosis* and Graded Exposure to Tuberculosis.

	Total				TB-Exposed Only			
	Exposure category based on TB contact score				Exposure category based on TB contact score			
	Odds Ratio (95% CI)				Odds Ratio (95% CI)			
	8–10	11–12	13–14	15–16	8–10	11–12	13–14	15–16
TST≥10 mm	ref	3.71 (1.22, 11.34)	5.50 (1.88, 16.08)	8.00 (2.10, 30.42)	ref	3.97 (1.19, 13.28)	4.40 (1.38, 14.08)	7.33 (1.67,32.21)
TST≥15 mm	ref	5.70 (1.16, 27.99)	6.79 (1.45, 31.80)	12.09 (2.31, 63.42)	ref	4.89 (0.98, 24.53)	4.09 (0.84,19.93)	7.50 (1.35, 41.72)
QFTGIT	ref	2.38 (0.46, 12.37)	6.60 (1.41, 30.89)	10.29 (1.97, 53.85)	ref	2.00 (0.38, 10.61)	3.64 (0.75,17.77)	7.50 (1.35, 41.71)
T-Spot. TB	ref	2.38 (0.46, 12.37)	5.75 (1.23, 26.99)	8.77 (1.68, 45.88)	ref	2.00 (0.38,10.61)	3.04 (0.62,14.95)	6.14 (1.10, 34.21)

Table 5 shows the concordance and discordance between the IGRAs and TST results at the two TST cut-off thresholds for the overall cohort and for TB-exposed and active TB cases at the baseline month 0 visit. For the overall cohort, concordance for positive IGRAs and positive TST ranged from 41.6–45.5% for the TST threshold of 10 mm and from 62.1–67.2% for the threshold of 15 mm. Concordance for negative IGRAs and negative TST ranged from 92.2–94.1% for the TST cut-off of 10 mm and was 89.4% for the TST threshold of 15 mm. Agreement based on the kappa statistic between IGRAs and TST at both cut-off values was higher among children with active TB compared with TB-exposed children. Agreement based on the kappa statistic was also higher with TST cut-off of ≥15 mm compared with ≥10 mm for the overall cohort and for both TB-exposed and active TB cases. Of the 87 TB-exposed children who had a TST≥10 mm, 41.4% had a positive QFNGIT result while 36.8% had a positive T-Spot.TB result. Among the 47 TB-exposed children who had a TST≥15 mm, QFNGIT positivity increased to 61.7% while T-Spot.TB positivity increased to 57.5%. Of the 14 children with active TB who had a TST≥10 mm, 71.4% had a positive QFNGIT result, with the same proportion having a positive T.Spot-TB result. Among the 11 children with active TB who had a TST≥15 mm, QFNGIT positivity increased to 90.9% while T-Spot.TB positivity increased to 81.8%. Concordance for positive QFNGIT and T-Spot.TB was 85.1% for the overall cohort, 83.3% for TB-exposed cases, and 90.9% for active TB cases.

**Table 5:** Concordance and Discordance between IGRAs and TST, Baseline Month 0 Visit.

	QFNGIT vs. TST					TSpot vs. TST					QFNGIT vs. TSpot				
	Concordant results		Discordant results			Concordant results		Discordant results			Concordant results		Discordant results		
	N (%)		N (%)			N (%)		N (%)			N (%)		N (%)		
	QFNGIT+	QFNGIT–	QFNGIT+	QFNGIT–	Kappa	TSpot+	TSpot–	TSpot+	TSpot–	Kappa	QFNGIT+	QFNGIT–	QFNGIT+	QFNGIT–	Kappa
	TST+	TST–	TST–	TST+	(95% CI)	TST+	TST–	TST–	TST+	(95% CI)	TSpot+	TSpot–	TSpot–	TSpot+	(95% CI)
Overall cohort															
TST≥10 mm	46/101 (45.5)	48/51 (94.1)	3/51 (5.9)	55/101 (54.5)	0.32 (0.21, 0.43)	42/101 (41.6)	47/51 (92.2)	4/51 (7.8)	59/101 (58.4)	0.27 (0.16, 0.38)	40/47 (85.1)	99/110 (90.0)	11/110 (10.0)	7/47 (14.9)	0.73 (0.62, 0.85)
TST≥15 mm	39/58 (67.2)	84/94 (89.4)	10/94 (10.6)	19/58 (32.8)	0.58 (0.45, 0.72)	36/58 (62.1)	84/94 (89.4)	10/94 (10.6)	22/58 (37.9)	0.54 (0.40, 0.68)	NA	NA	NA	NA	NA
TB-exposed															
TST≥10 mm	36/87 (41.4)	42/44 (95.5)	2/44 (4.6)	51/87 (58.6)	0.29 (0.18, 0.40)	32/87 (36.8)	41/44 (93.2)	3/44 (6.8)	55/87 (63.2)	0.23 (0.12, 0.34)	30/36 (83.3)	90/100 (90.0)	10/100 (10.0)	6/36 (16.7)	0.71 (0.58, 0.84)
TST≥15 mm	29/47 (61.7)	75/84 (89.3)	9/84 (10.7)	18/47 (38.3)	0.53 (0.38, 0.69)	27/47 (57.5)	76/84 (90.5)	8/84 (9.5)	20/47 (42.6)	0.51 (0.35, 0.66)	NA	NA	NA	NA	NA
Active TB															
TST≥10 mm	10/14 (71.4)	6/7 (85.7)	1/7 (14.3)	4/14 (28.6)	0.52 (0.16, 0.87)	10/14 (71.4)	6/7 (85.7)	1/7 (14.3)	4/14 (28.6)	0.52 (0.16, 0.87)	10/11 (90.9)	9/10 (90.0)	1/10 (10.0)	1/11 (9.1)	0.81 (0.56, 1.00)
TST≥15 mm	10/11 (90.9)	9/10 (90.0)	1/10 (10.0)	1/11 (9.1)	0.81 (0.56, 1.0)	9/11 (81.8)	8/10 (80.0)	2/10 (20.0)	2/11 (18.2)	0.62 (0.28, 0.96)	NA	NA	NA	NA	NA

+Positive.

–Negative.

TST: Tuberculin skin test.

TST positive: TST≥10 mm or ≥15 mm.

TST negative: TST<10 mm or <15 mm.

QFNGIT: QuantiFERON Gold In-tube.

TSpot: T-Spot.TB.

CI: Confidence interval.

Table 6 displays the bivariable and multivariable logistic regression results for the following outcomes: (1) having a positive QFNGIT result, (2) having a positive T-Spot.TB result, (3) having a positive QFNGIT and a positive T-Spot.TB result, (4) having a TST≥10 mm, and (5) having a TST≥15 mm. In multivariable analysis, having had exposure to a primary and secondary caregiver in the household with TB (OR = 4.07, 95% CI 1.38–11.99 for primary caregiver, OR = 3.95, 95% CI 1.25–12.52 for secondary caregiver vs. relative or other contact in the household) was significantly associated with having a positive QFNGIT result. In multivariate analysis, having a higher TB contact score (OR = 3.15, 95% CI 1.35–7.34) and having active TB (OR = 3.03, 95% CI 1.11–8.29 vs. TB-exposed) were associated with having a positive T-Spot.TB result. Having active TB (OR = 3.65, 95% CI 1.12–11.94 vs. TB-exposed) was significantly associated with having both a positive T-Spot.TB and a positive QFNGIT result. Older age (OR = 1.14, 95% CI 1.03–1.26) was associated with having a positive TST≥10 mm, while older age (OR = 1.13, 95% CI 1.03–1.25) and having a primary caregiver in the household with TB (OR = 3.59, 95% CI 1.34–9.59) were associated with having a positive TST≥15 mm. Other variables such as HIV status and weight-for-age Z-score were not significantly associated with any of the IGRA and TST outcomes.

**Table 6:** Association between Covariates of Interest and Response to IGRAs and TST.

	Positive QFNGIT		Positive TSpot.TB		Positive QFNGIT and Positive TSpot.TB		Positive TST≥10 mm		Positive TST≥15 mm	
	Bivariate	Multivariate	Bivariate	Multivariate	Bivariate	Multivariate	Bivariate	Multivariate	Bivariate	Multivariate
Age (continuous)	1.04 (0.96, 1.12)	-	1.01 (0.93, 1.10)	-	0.99 (0.91, 1.08)	-	1.11 (1.01, 1.21)	1.14 (1.03, 1.26)	1.08 (1.10, 1.21)	1.13 (1.03, 1.25)
Gender										
Female	1.02 (0.52, 1.99)	-	0.90 (0.45, 1.78)	-	1.07 (0.52, 2.20)	-	1.43 (0.72, 2.82)	-	1.15 (0.60, 2.20)	-
Male	reference		reference		reference		reference		reference	
History of prior active or latent TB										
Yes	1.96 (0.21, 18.00)	-	NA	-	NA	-	8.60 (0.94, 79.00)	-	NA	-
No	reference						reference			
TB index case relationship to child^a^										
Primary caregiver in household with TB	3.25 (1.36, 7.77)	4.07 (1.38, 11.99)	1.55 (0.68, 3.53)	-	2.90 (1.14, 7.40)	2.89 (0.94, 8.89)	1.44 (0.61, 3.41)	-	2.36 (1.06, 5.23)	3.59 (1.34, 9.59)
Secondary caregiver in household with TB	3.95 (1.50, 10.43)	3.95 (1.25, 12.52)	1.76 (0.70, 4.47)		3.58 (1.28, 9.99)	2.80 (0.85, 9.25)	0.87 (0.34, 2.23)		1.70 (0.68, 4.25)	2.11 (0.70, 6.36)
Relative/other contact in household with TB	reference	reference	reference		reference	reference	reference		reference	reference
Type of exposure in household with TB										
Sleeps in same room	reference	reference	reference	-	reference	reference	reference	-	reference	-
Lives/sleeps in same or different house	0.43 (0.22, 0.86)	0.62 (0.22, 1.74)	0.52 (0.26, 1.04)		0.41 (0.19, 0.85)	0.73 (0.25, 2.09)	0.50 (0.25, 1.01)		0.56 (0.29, 1.09)	
Duration of average contact per day with TB index case										
0–7 hours	0.57 (0.25, 1.27)	-	0.45 (0.19, 1.06)	-	0.60 (0.25, 1.45)	-	0.44 (0.21, 0.92)	0.62 (0.26, 1.46)	0.56 (0.26, 1.21)	-
8+ hours	reference		reference		reference		reference	reference	reference	
Duration of contact with TB index case in last 12 months										
≤7 months	0.51 (0.26, 1.01)	0.68 (0.29, 1.60)	0.49 (0.24, 0.97)	0.80 (0.37, 1.73)	0.53 (0.26, 1.09)	-	0.49 (0.24, 1.00)	-	0.83 (0.43, 1.62)	-
>7 months	reference	reference	reference	reference	reference		reference		reference	
Index TB case history										
Sputum acid fast smear negative	1.03 (0.30, 3.60)	-	0.44 (0.09, 2.09)	-	0.56 (0.12, 2.66)	-	0.42 (0.09, 2.03)	-	1.74 (0.44, 6.82)	-
Sputum acid fast smear positive	reference		reference		reference		reference		reference	
Mycobacterium TB culture positive	1.23 (0.23, 6.54)	-	1.08 (0.20, 5.78)	-	2.13 (0.25, 18.23)	-	1.00 (0.18, 5.65)	-	1.60 (0.30, 8.53)	-
Mycobacterium TB culture negative	reference		reference		reference		reference		reference	
TB contact score										
8 to 12	reference	reference	reference	reference	reference	reference	reference	reference	reference	reference
13+	4.04 (1.81, 8.99)	1.98 (0.64, 6.11)	3.50 (1.57,7.81)	3.15 (1.35, 7.34)	4.35 (1.76, 10.71)	2.37 (0.74, 7.58)	2.59 (1.28, 5.23)	2.21 (0.99, 4.98)	2.19 (1.09, 4.43)	0.83 (0.35, 1.99)
HIV status										
HIV negative	0.42 (0.03, 6.06)	-	0.75 (0.05, 11.65)	-	0.44 (0.03, 7.67)	-	1.67 (0.12, 24.26)	-	1.14 (0.08, 16.95)	-
Unknown status/not tested	0.23 (0.02, 2.57)		0.88 (0.08, 9.94)		0.71 (0.06, 8.08)		4.57 (0.40, 51.80)		1.29 (0.11, 14.53)	
HIV positive	reference		reference		reference		reference		reference	
Weight for age Z-score (continuous)	0.87 (0.66, 1.14)	-	0.88 (0.67, 1.16)	-	0.85 (0.63. 1.14)	-	0.84 (0.64, 1.10)	-	0.89 (0.69, 1.16)	-
Summary of child’s TB diagnosis										
Active TB	2.64 (1.04, 6.71)	2.94 (0.87, 9.93)	3.06 (1.20, 7.80)	3.03 (1.11, 8.29)	3.21 (1.25, 8.28)	3.65 (1.12, 11.94)	1.00 (0.38, 2.66)	0.88 (0.31, 2.54)	1.93 (0.76, 4.86)	-
TB-exposed	reference	reference	reference	reference	reference	reference	reference	reference	reference	

a Non-household contact with TB was excluded from the models due to 0 cells.

NA: not applicable.

## Discussion

In this first study directly comparing two IGRAs to TST in 158 children with recent exposure to an adult index case with active TB in Thailand, we found that QFNGIT and T-Spot.TB performed well without any indeterminate or equivocal/borderline results. Past studies have shown that the proportion of children with indeterminate results for IGRAs varied widely from 0–40%, with higher proportions reported in children who were younger in age, were malnourished, or had immunodeficiency or other acute infections. [19]–[23] About two-thirds of our overall cohort had a TST≥10 mm induration and 38.6% had a TST≥15 mm induration, with no significant difference by TB infection status. We observed that in the overall cohort and separately for TB-exposed and active TB cases, QFNGIT and T-Spot.TB positivity (32.5% and 29.9%, respectively) was lower than TST at both cut-off thresholds. This finding is similar to a study among South African school children with household TB contact that observed 33.2% of the children having a positive QFNGIT compared with 42.5% having a positive TST. Our test positivity rates differ from other studies. In a study evaluating QFNGIT and TST among children in Papua New Guinea, [15] QFNGIT was more likely to be positive than TST in children with probable and possible TB (34.2% vs. 24.9%). Compared to our study cohort, the Papua New Guinea cohort was younger in age, had more children who were nutritionally deficient as defined by weight-for-age Z-score, and had more HIV-infected children; such differences might explain the disparity in IGRA and TST positivity levels between the two studies.

Our study found that both the IGRAs and TST at the two cut-off thresholds performed well with increasing TB exposure gradient. In addition, we did not detect any significant differences between the performances of the tests with increasing TB exposure, consistent with a meta-analysis by Mandalakas et al comparing IGRAs with TST in TB-exposed children. [8] In a Brazilian study among young children with recent TB exposure, a linear, positive association was also noted between both QFNGIT and TST and level of TB exposure. [24] Based on our finding of a lack of superiority of the IGRAs over the TST with increasing TB exposure, it appears that utilizing TST, as currently recommended by the Thai national TB guidelines, would be a reasonable alternative to the IGRAs, in diagnosing latent and active TB infections in Thai children who match our study population (i.e., those who are generally healthy without any underlying immune compromise and who have previously received BCG vaccinations). Importantly, the TST is widely accessible and affordable compared to IGRAs, which also require operational capacity (e.g., laboratory infrastructure), and may be more appropriate for Thailand and other resource-limited settings.

Our study noted that both QFNGIT and T-Spot.TB positivity was higher in children with active TB compared with TB-exposed (55.0% vs. 29.2% for QFNGIT and 55.0% vs. 26.3% for T-Spot.TB). No statistically significant difference in TST positivity at the two cut-off thresholds, however, was seen between active and TB-exposed cases. Compared with TB-exposed children, children with active TB were 4.2 to 5.8 times more likely to have a positive QFNGIT result, to have a positive T-Spot TB result, and to have both positive QFNGIT and T-Spot TB results. Prior studies have documented higher sensitivity of IGRAs compared with TST in children with active TB. [11], [15] However, in a United Kingdom study in 209 children, the sensitivity of TST was 83% for culture-confirmed, active TB, comparable to 80% for QFNGIT and higher than 58% for T-Spot.TB [25].

In our study, we detected variable levels of agreement between QFNGIT, T-Spot.TB, and TST at the two cut-off thresholds, which is consistent with other studies that noted discrepancies between IGRA and TST results among children in both low and high TB incidence settings. [3], [12], [13], [23], [26] Levels of agreement between QFNGIT and TST and between T-Spot.TB and TST were low for our overall cohort, with kappa ranging from 0.32–0.58 and 0.27–0.54, respectively, although higher levels of agreement were seen with the 15 mm threshold compared with 10 mm and with active TB compared with TB-exposed cases. Similar to a previous study, [22] many of the discordant results were due to a QFNGIT−/TST+ or T-Spot.TB−/TST+, particularly with the TST cut-off of 10 mm. This high rate of false positive TST result may be attributable to high background prevalence of BCG vaccination in our study population (as well as in the general Thai pediatric population). The prevalence of nontuberculous mycobacteria, which can affect the false positivity rate of TST, is unknown in Thai children. Agreement between QFNGIT and T-Spot.TB ranged from 0.71 to 0.81, comparable to previous reports of agreement between the two IGRAs in children. [25]–[27] We found that among TB-exposed children who had a TST≥10 mm, 41.4% had a positive QFNGIT result and 36.8% had a positive T-Spot.TB result. If IGRAs, rather than TST with a cut-off of 10 mm, are used to guide who in the healthy Thai pediatric population to start INH prophylaxis for latent TB, it would reduce the number of children considered for preventive treatment by approximately 60%, thereby potentially limiting the number of children who may experience adverse medication-related side effects.

Our study finding that children with active TB were more likely than TB-exposed children to have been exposed to a secondary caregiver in the household with TB and less likely have been exposed to a primary caregiver or relative or other contact in the household with TB compared with TB-exposed children may appear counterintuitive. It is possible that the active TB children were exposed to TB from secondary caregivers such as grandparents or household helpers, who might spend more time with the children during the day (but not overnight), rather than from primary caregivers such as parents. It is also possible that primary caregivers with active TB might also be more likely to have initiated anti-tuberculosis drugs and have decreased infectivity than secondary caregivers with active TB. Designation of primary and secondary caregiver might not have been consistent across all participants. It is important to note that although we found a difference with type of caregiver, there was no significant difference in type of exposure, duration of average contact per day, and duration of contact in the last 12 months to the index TB case between active and TB-exposed children. In our multivariable analysis, children who had exposure to a primary and secondary caregiver in the household with TB were 3.5 to 4.3 times more likely, respectively, to have a positive QFNGIT result, while those with a higher mean TB contact score were 3.15 times more likely to have a positive T-Spot.TB. In addition, children who had exposure to a primary caregiver in the household with TB were 3.59 times more likely to have a TST≥15 mm, but not TST≥10 mm. Our results are consistent with studies that showed a strong correlation between positivity of IGRAs (as well as TST at three different cut-off thresholds) and a high TB exposure gradient. [8], [28]–[30] Older age was found to be associated with having a positive TST at both threshold levels, but not with a positive QFNGIT or T-Spot.TB. Nutritional status, as measured by weight-for-age Z-score, and HIV status were not associated with a positive IGRA or TST, with the latter finding most likely due to the small sample of children in our study with HIV infection. Our findings differed from previous reports of IGRA and TST results being affected by age, HIV status, and nutritional status [31]–[33].

Our study has several limitations. Our study’s direct comparison of IGRAs to TST involved comparing the performances of the tests for increasing TB exposure gradient. As in other studies, our analysis was complicated by the lack of gold standard in diagnosing TB. [11], [34] In addition, our small sample of active TB cases (20 children) and high burden of childhood TB in Thailand limited any sensitivity and specificity analyses using active TB as a surrogate reference due to bias that could be arise from these conditions. Our study only enrolled 3 HIV-infected children, precluding a direct comparison of the IGRAs and TST by HIV status. In addition, most of the children in our study who were otherwise healthy, had no history of maternal positive HIV serostatus, and had no HIV-related signs and symptoms were presumed to be HIV-negative and did not receive HIV testing, which might have resulted in misclassification bias. Strengths of our study include the incorporation of both IGRAs (QFNGIT and T-Spot.TB) to compare to TST at two different cut-off thresholds, our use of extensive clinical, diagnostic, and microbiologic parameters in determining or excluding active TB infection status, overall excellent performance of the IGRAs with no indeterminate or equivocal/borderline results, and very low rate of incomplete IGRA testing by study participants (only 1 child did not complete the IGRAs at the baseline visit).

## Conclusions

In summary, both QFNGIT and T-Spot.TB performed well in our generally healthy Thai pediatric study population with recent exposure to adults with active pulmonary TB, with no indeterminate or equivocal/borderline results. We did not find any significant differences between the performances of the IGRAs and TST at the two cut-offs with increasing TB exposure. Using TST, as is currently recommended in the Thai national TB guidelines, may be more ideal than IGRAs in diagnosing latent and active TB in healthy Thai children given the lack of difference in test performance between the diagnostic tests noted in our study and given cost constraints (TST is considerably less expensive than IGRAs) and logistics, with TST not requiring any laboratory infrastructure. However, TST does have other operational considerations, such as inter-reader variability and the need for two separate visits for TST implant and reading, that the IGRAs do not have that may make using the IGRAs in TB diagnosis appealing.
